# Genome-wide expansion and reorganization during grass evolution: from 30 Mb chromosomes in rice and Brachypodium to 550 Mb in *Avena*

**DOI:** 10.1186/s12870-023-04644-7

**Published:** 2023-12-08

**Authors:** Qing Liu, Lyuhan Ye, Mingzhi Li, Ziwei Wang, Gui Xiong, Yushi Ye, Tieyao Tu, Trude Schwarzacher, John Seymour (Pat) Heslop-Harrison

**Affiliations:** 1 Key Laboratory of Plant Resources Conservation and Sustainable Utilization, Guangdong Provincial Key Laboratory of Applied Botany, South China Botanical Garden, Chinese Academy of Sciences, Guangzhou, 510650 China; 2 South China National Botanical Garden, Guangzhou, 510650 China; 3 Center for Conservation Biology, Core Botanical Gardens, Chinese Academy of Sciences, Guangzhou, 510650 China; 4 University of Chinese Academy of Sciences, Beijing, 100049 China; 5 Bio&Data Biotechnologies Co. Ltd, Guangzhou, 510663 China; 6 Henry Fok School of Biology and Agriculture, Shaoguan University, Shaoguan, 512005 China; 7 Department of Genetics and Genome Biology, Institute for Environmental Futures, University of Leicester, Leicester, LE1 7RH UK

**Keywords:** Ancestral karyotype, *Avena*, Chromosomal rearrangements, Genomic expansion, Oat, Retrotransposons, Structural variation, Translocations

## Abstract

**Background:**

The BOP (Bambusoideae, Oryzoideae, and Pooideae) clade of the Poaceae has a common ancestor, with similarities to the genomes of rice, *Oryza sativa* (2*n* = 24; genome size 389 Mb) and Brachypodium, *Brachypodium distachyon* (2*n* = 10; 271 Mb). We exploit chromosome-scale genome assemblies to show the nature of genomic expansion, structural variation, and chromosomal rearrangements from rice and Brachypodium, to diploids in the tribe Aveneae (e.g., *Avena longiglumis*, 2*n* = 2*x* = 14; 3,961 Mb assembled to 3,850 Mb in chromosomes).

**Results:**

Most of the *Avena* chromosome arms show relatively uniform expansion over the 10-fold to 15-fold genome-size increase. Apart from non-coding sequence diversification and accumulation around the centromeres, blocks of genes are not interspersed with blocks of repeats, even in subterminal regions. As in the tribe Triticeae, blocks of conserved synteny are seen between the analyzed species with chromosome fusion, fission, and nesting (insertion) events showing deep evolutionary conservation of chromosome structure during genomic expansion. Unexpectedly, the terminal gene-rich chromosomal segments (representing about 50 Mb) show translocations between chromosomes during speciation, with homogenization of genome-specific repetitive elements within the tribe Aveneae. Newly-formed intergenomic translocations of similar extent are found in the hexaploid *A*. *sativa*.

**Conclusions:**

The study provides insight into evolutionary mechanisms and speciation in the BOP clade, which is valuable for measurement of biodiversity, development of a clade-wide pangenome, and exploitation of genomic diversity through breeding programs in Poaceae.

**Supplementary Information:**

The online version contains supplementary material available at 10.1186/s12870-023-04644-7.

## Background

Genomic studies have shed light on the nature and processes of gene evolution, with variation in DNA and RNA sequence data enabling development of robust phylogenies [1]. Multiple polyploidy or whole-genome duplication (WGD) events have played a major part in plant speciation and genome evolution [2, 3] with the separation of Poales from other monocotyledonous orders around 60–110 million years ago (Mya) [4]. The ρ WGD event occurred 50–70 Mya, at the end of the Cretaceous period [5–7] and marked separation of the BOP (Bambusoideae, Oryzoideae, and Pooideae) clade, which includes rice, oats, and wheat, from other grass lineages [8–11]. In contrast to other angiosperm families such as Brassicaceae [12, 13], further WGD events in the BOP clade occurred much more recently [14], and many polyploids in both Triticeae (wheat) and Aveneae (oats) arose in the last few million years [15]. Based on palaeogenomic research, Murat et al. (2010) proposed an ancestral grass karyotype (AGK) based on 5 to 7 chromosomes, with the post-ρ WGD karyotype having 12 chromosome pairs, similar to extant *Oryza sativa* (rice, 2*n* = 2*x* = 24) and with derived numbers down to *n* = 5 (*Brachypodium distachyon*, Brachypodium, 2*n* = 2*x* = 10) [16]. Rice has preserved the AGK [6], and also like Brachypodium, has a small genome size with neither the transposon activities nor the repeat accumulation observed elsewhere in the grasses. Genome size shows very substantial variation in the BOP clade, from 271 Mb in Brachypodium and 389 Mb in rice [17, 18], to more than 4,000 Mb in many diploid Triticeae and a similar size in Aveneae (both *x* = 7) [19, 20]. Genome variation and chromosome reorganization have been shown to be important in plant breeding [2–4]. Further work is needed to understand the interplay between repetitive DNA proliferation, insertion/retention bias in the BOP clade, and how to harness this biology to enhance traits of agronomic importance.

For larger genomes, genome-scale analyses of evolution have been hampered by sequence mis-assembly in scaffolds (and linkage breakage), fragmented assemblies, as well as collapse of similar reads of repetitive motifs from thousands of copies to a small number during assembly [21, 22]. Long-read sequencing technologies (e.g., from Oxford Nanopore Technologies, ONT, or PacBio) combined with genome scaffolding methods (e.g., high-throughput chromatin conformation capture, Hi-C) now enable inclusion of the majority of repetitive DNA sequences in contiguous genome assemblies that reach chromosome-scale [23–26].

Here we aimed to characterize the nature of genome-size expansion within the grass BOP clade, from the small genomes of rice and Brachypodium, represented as being close to the AGK, to the magnitude larger genomes of diploid *Avena* species, integrating conserved synteny and chromosome-block evolution. In addition, we addressed whether there are genomic hot-spots of integration of repetitive elements, and whether genes remain in compact blocks during genomic expansion. Our results offer genomic insights into the evolutionary history of the Pooideae grasses and the basis for developing the grass pangenome.

## Materials and methods

### Genome sequences

A chromosome-scale genome assembly of *Avena longiglumis* (ALO; PI 657387; US Department of Agriculture at Beltsville, https://www.ars-grin.gov/, originally collected in Morocco) are deposited into the National Center for Biotechnology Information Sequence Read Archive (NCBI SRA) under accession number PRJNA956334. The chromosome-scale genome assemblies of *A. atlantica* (AAT) and *A. eriantha* (AER) were downloaded from https://genomevolution.org/coge/GenomeInfo.pl?gid=53337 and https://genomevolution.org/coge/GenomeInfo.pl?gid=53381, respectively [20]. A chromosome-scale genome assembly of *Brachypodium distachyon* (BDI) was downloaded from NCBI SRA (PRJNA32607) [17]. A chromosome-scale genome assembly of *Oryza sativa* (OSA) was downloaded from https://phytozome-next.jgi.doe.gov/info/Osativa_v7_0 [18].

### Genome annotation

#### Repeat analysis

*De novo* repeat prediction was carried out across all the reference genomes. We found that the use of published annotations from different versions of software resulted in unsatisfactory comparisons of identifications and abundances of repetitive elements. For the ALO assembly, repeat prediction was carried out by EDTA v.1.7.0 (Extensive *de-novo* TE Annotator [27], which was composed of eight software modules. The LTRharvest (LTRharvest, PRID:SCR_018970) [28], LTR_FINDER_parallel (LTR_FINDER, PRID:SCR_015247) [29], LTR_retriever [27], Generic Repeat Finder [30] and TIR-Learner [31] modules were included to identify TIR transposons. The HelitronScanner v.1.0 [32] module was used to identify *Helitron* transposons. The RepeatModeler v.2.0.2a [33] module was used to identify TEs (such as *LINEs*). Finally, the RepeatMasker v.4.1.1 (RepeatMasker, RRID:SCR 012954) [34] module was used to annotate fragmented TEs based on homology to structurally annotated TEs. In addition, the TEsorter v.1.1.4 [35] module was used to identify TE-related genes (Additional file 2: Table S8). The final set of repetitive sequences in the ALO assembly was obtained by integrating *ab initio-*predicted TEs and those identified by homology through RepeatMasker (Additional file 2: Table S8A). Intact LTR-RTs were identified using LTR_retriever [28]. For comparison, the same repeat analysis protocol was applied to other two grass genomes, *B*. *distachyon* [17] and *O*. *sativa* [18] (Additional file 2: Table S8B), in the context of genome size. All LTR-RT families were clustered based on their LTR sequences.

**Fig. 1:**
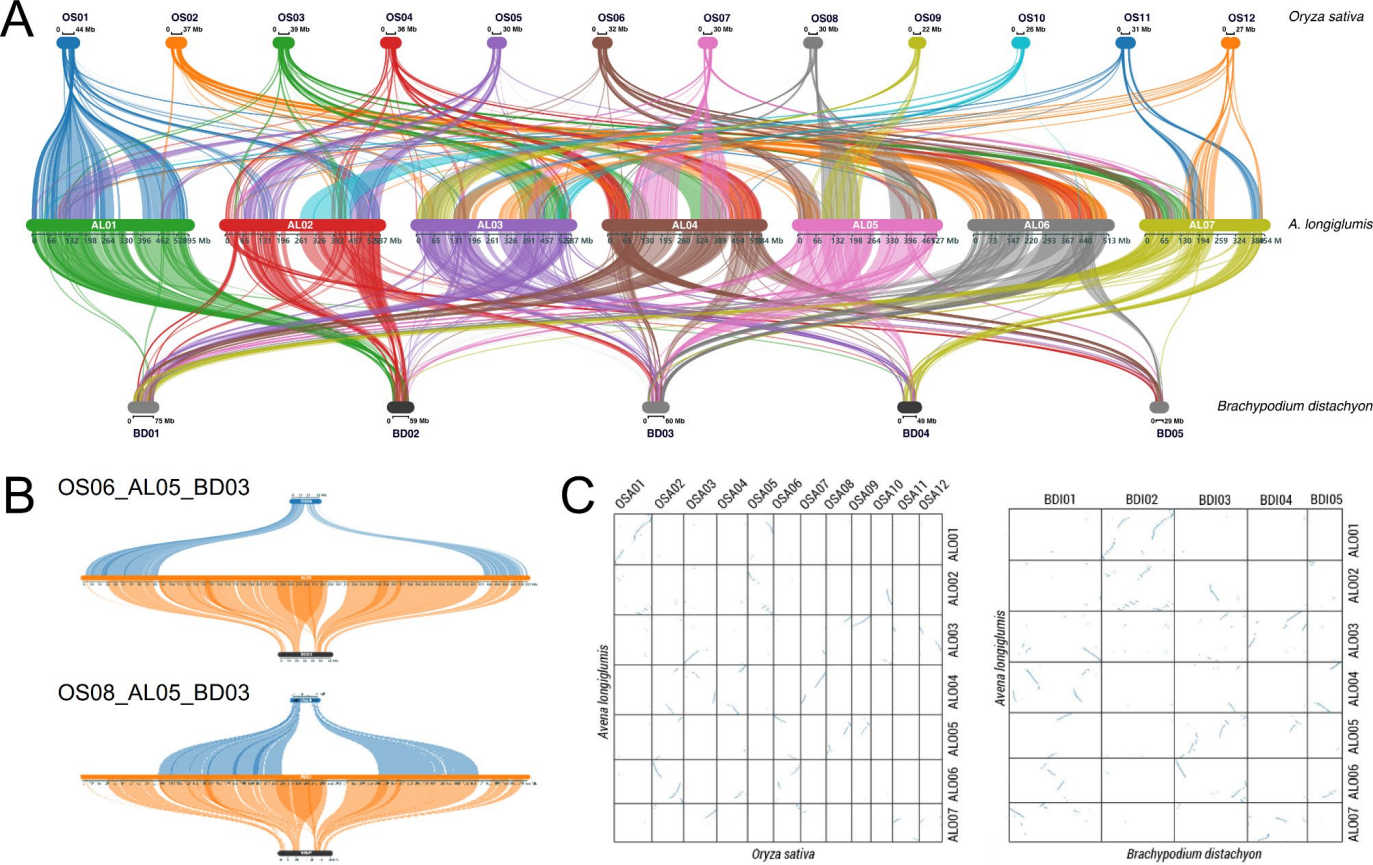
Deep syntenic relationship of *Oryza sativa* (OSA), *Avena longiglumis* (ALO), and *Brachypodium distachyon* (BDI) chromosomes, drawn to scale, showing detailed conservation of syntenic blocks and the genomic expansion between OSA (*x* = 12; 373 Mb), BDI (*x* = 5; 271 Mb) and ALO (*x* = 7; 3,850 Mb). **A** Syntenic analysis of OSA (top), ALO (middle), and BDI (bottom). Subterminal regions are frequently involved in interspecific evolutionary translocations. **B** Syntenic analyses of chromosomes OS06-AL05-BD03 (top) and OS08-AL05-BD03 (bottom). **C** Dotplots (not to scale) of OSA-ALO (left) and BDI-ALO (right) genomes

Centromere locations in ALO were identified by the following genomic features: (1) high abundances of repeat sequences on chromosome dotplots (Additional file 1: Figure S3); (2) discontinuities in the Hi-C contact map (Figure S4 in Liu et al. [36]); (3) locations of barley (*Hordeum vulgare*) *Gypsy* LTR *Cereba* (KM948610) [37] sequence used to identify centromeres in wheat (*Triticum aestivum*, TAE) [38] [the *Cereba* sequence was aligned to the ALO assembly using BLASTN and the centromere cores were identified using Geneious Prime v.2021.1.1 (https://www.geneious.com/; Additional file 2: Table S5B)]; (4) SynVisio [39] visualization of gaps and conserved regions between the ALO and OSA assemblies (Fig. 1, Additional file 2: Table S5C); and regions of low gene density along each ALO chromosome. Centromeric cores were defined by overlapping high-abundance repeat regions on ALO chromosome dotplots and regions of low gene density on ALO chromosomes.

#### Gene family identification and phylogenetic tree reconstruction

To evaluate evolution and divergence of the genome assembly, protein-coding gene sequences from six species, ALO, AAT [20], AER [20], *A*. *strigosa* (AST) [40], BDI [17], and OSA [18], were downloaded from Phytozome v.13 [41] and NCBI (https://www.ncbi.nlm.nih.gov/) for comparative analyses (Additional file 2: Tables S3 and S4). When one gene had multiple transcripts, only the longest transcript in the coding region was kept for further analysis. Paralogs and orthologs were clustered with OrthoFinder v.2.3.14 [42], using standard parameters, with Diamond v.0.9.24 [43]. Single-copy of orthologous genes were extracted from OrthoFinder [42].

#### Orthogroup analysis

**Fig. 2:**
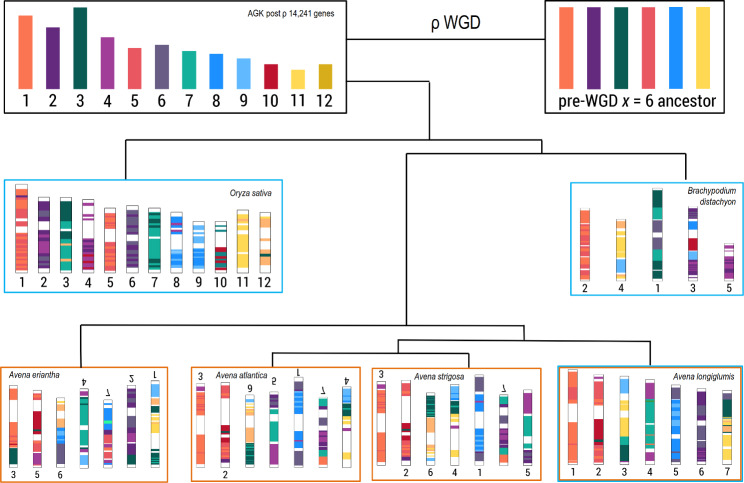
Reconstruction of ancestral chromosomes for the six species showing conservation of major syntenic blocks from the ancestral grass karyotype (AGK), with fusions and insertions leading to the reduced chromosome numbers (some are upside down to display features of evolutionary conservation). Genes from the ancestral linkage groups are indicated by different colors, with pairs of similar colors representing the pre-ρ whole-genome duplication (WGD)

We used standard methods to discover syntenic blocks of genes [44, 45]. Protein sequences within and between genomes were searched against one another to detect putative homologous genes (*E* value < 1e-5) using BLASTP. With homologous gene data as input, MCScanX [46] was used to infer homologous blocks involving collinear genes within and between genomes. The maximum gap length between collinear genes along a chromosome region was set to 50 genes [47]. Homology dotplots were constructed using SynVisio [39] to reveal genomic correspondence in ALO, between three *Avena* species, between ancestral grass karyotype (AGK) and seven grass species, and between ALO, OSA, and BDI (Fig. 2, Additional file 2: Table S6).

### Fluorescence *in situ* hybridization

Seeds of the hexaploid oat *A. sativa* (2*n* = 6*x* = 42) were used for chromosome preparations. The plant materials and probes [AF226603_45bp (labeled with tetrachloro-fluorescein TET), pAs120a (labeled with biotin) and Ab-T148 (labeled with digoxingenin)] used in this experiment were as reported in Liu et al. [48]. Root tips were fixed in 96% ethanol: glacial acetic acid (3:1) for at least 1.5 h and stored in the fixative at − 20 °C overnight. An enzyme solution containing 0.2% Cellulase Onozuka R10 (Yakult Pharmaceutical, Tokyo), 2% Cellulase C1184 (Sigma-Aldrich, St Louis, USA), and 3% Pectinase (P4716; Sigma-Aldrich, St Louis, USA) was used to digest root tips for 90 min at 37 °C. Finally, root tips were macerated in a drop of 60% acetic acid, and squashed gently under a coverslip.

Fluorescence in situ hybridization (FISH) was performed as described by Liu et al. [48] and Schwarzacher and Heslop-Harrison [49]. The hybridization mixture (strigency 76%), containing 50% formamide, 2 × SSC (Saline Sodium Citrate buffer; 0.3 M NaCl, 0.03 M sodium citrate), 10% dextran sulphate, 0.125% SDS (sodium dodecyl sulphate), 0.125mM EDTA (ethylenediamine-tetraacetic acid), 1 µg sheared salmon sperm DNA, and 100 ng of labeled probes, were applied to each slide. After hybridization at 37°C overnight in a ThermoHybaid HyPro-20, slides were washed in 0.1 × SSC at 42°C. FISH probe hybridization sites were detected via fluorescein isothiocyanate (FITC) conjugated anti-digoxigenin (200 µg/ml; Roche Diagnostics), and streptavidin Alexa Fluor 647 (Molecular Probes, Invitrogen). Slides were counterstained with 4’, 6-diamidino-2-phenylindole (DAPI; 3 mg/ml)-antifade solution (AF1, Citifluor, London, UK; 50%). FISH images were captured by a Nikon Eclipse 80i epifluorescent microscope fitted with appropriate sets of band-pass filters, a DS-QiMc monochromatic camera, and NIS-Elements v.2.34 (Nikon, Tokyo, Japan). For each metaphase, four 1280 × 1024 pixel size, single channel (pseudo-colored, yellow, red, green, and blue respectively) images were analyzed using Image J v.1.51j8 (Wayne Rasband, NIH, USA) and superimposed in Photoshop CS6 v.13.0 (Adobe System, San Jose, CA, USA).

## Results

To understand the chromosomal structural variation, including translocations, duplications, and deletions, we examined the extent of chromosomal rearrangements in Pooideae species by examining gene synteny, gene locations on chromosomes, and the interspersion of non-coding repetitive DNA sequences. From the BOP clade, two species with small genome sizes, *O*. *sativa* Nipponbare [18] and *B*. *distachyon* [19], were compared with a new assembly of the diploid oat *A*. *longiglumis* (Additional file 2, Tables S1 and S2) [36], and three other diploid *Avena* species, *A*. *atlantica* [20], *A. eriantha* [20], and *A*. *strigosa* [40]. From a total of 19,954 gene families identified in ALO, about 10% (1,880) were analyzed as orthologous single-copy genes in AAT, AER, AST, as well as BDI and OSA (Additional file 2: Tables S3 and S4).

### Conserved synteny and genomic expansion in the BOP clade: *Avena*, rice, and brachypodium

The orthologous genes were used to generate a synteny (McScanX visualized by SynVisio [39, 46]) plot with chromosomes of the diploid species OSA, BDI, and ALO drawn to scale, showing lines linking each conserved group of genes between the species (Fig. 1, Additional file 1: Figure S1). Notably, the dense concentration of synteny lines in the 23–75 Mb chromosomes of OSA and BDI were spread throughout the orthologous 454–595 Mb chromosomes of ALO, apart from the centromeric regions (Fig. 1A). There were few larger gaps along the chromosome arms; and syntenic orthologous genes extended to the subterminal regions of all chromosomes. Figure 1B highlights chromosome AL05, which is largely syntenic to BD03. The distal regions of both chromosome arms are syntenic to OS06 while the central region is syntenic to OS08 (all syntenic relationships between ALO chromosomes shown in Additional file 1: Figure S2). Dotplots (not to scale, Fig. 1C) display large stretches of homologies in straight lines to the chromosome ends (into corners of the plots), supporting that syntenic regions of orthologous genes continue to the ends of chromosomes and emphasizing that there are minimal syntenic regions in broad centromeric regions.

The centromeric regions of ALO have a very low density of single-copy genes, but the *Cereba*-like retrotransposon is abundant (Additional file 1: Figures S3, S4 and Additional file 2: Table S5B) [36]. The regions adjacent to centromeres with lower gene density (large gaps in the SynVisio plots, Fig. 1A) share gene synteny with BDI in some chromosomes, but these genes appear to lack groups of conserved OSA orthologues on chromosomes AL01, AL04, AL05 and AL07 (visualized as no lines in the expanded region around the centromere of ALO chromosomes linked to OSA chromosomes) (Fig. 2, Additional file 1: Figure S2). In the comparison of AL04 and BD01 (Additional file 1: Figure S2N and P), the centromeric gap is translocated. In effect, genes flanking one side of the gap have moved to the other side of centromere between the two species, while the orthologous genes flanking the centromere are missing in OSA03 and OS07.

### Ancestral chromosome rearrangement and evolution

The proposed post-ρ AGK [5, 14] has 12 proto-chromosomes, here designated AG01 to AG12, with extensive similarities with rice. We mapped the AGK genes to chromosomes of ALO and five BOP grass species (AAT, AST, AER, BDI, and OSA). The corresponding regions of chromosomes, mosaic synteny blocks, were designed by different colors (Fig. 2, Additional file 2: Table S6). Features of the ancient ρ duplication are shown by chromosome pairs with similar shades of color (Fig. 2), and also shown by dot blots and some syntenic lines being duplicated (Additional file 1: Figure S5). The ancestral synteny blocks defined by shared gene sequences (Fig. 1) were conserved among the analyzed grasses, with distinct rearrangements involving translocations and fusions of syntenic blocks between species. Some rearrangement events are shared between all *x* = 5 and *x* = 7 species (e.g., the fusion of AG09 and AG11; or AG02 and AG03; both are seen in BDI and *Avena* species) or between the *x* = 7 species (AG12 and AG06 giving AL07; Fig. 2, Additional file 2: Table S6). Some evolutionary events associated with the 12 ancestral AGK chromosomes involve fusion and rearrangement of syntenic blocks, but it is notable that three events are characterized by insertion (nesting) of one chromosome into another chromosome. Especially, AL04 has AG07 inserted into AG04, AL06 has much of AG06 inserted into AG02, and AL05 has AG08 inserted into AL06 (Fig. 2).

### Chromosomal rearrangements within *Avena*

**Fig. 3:**
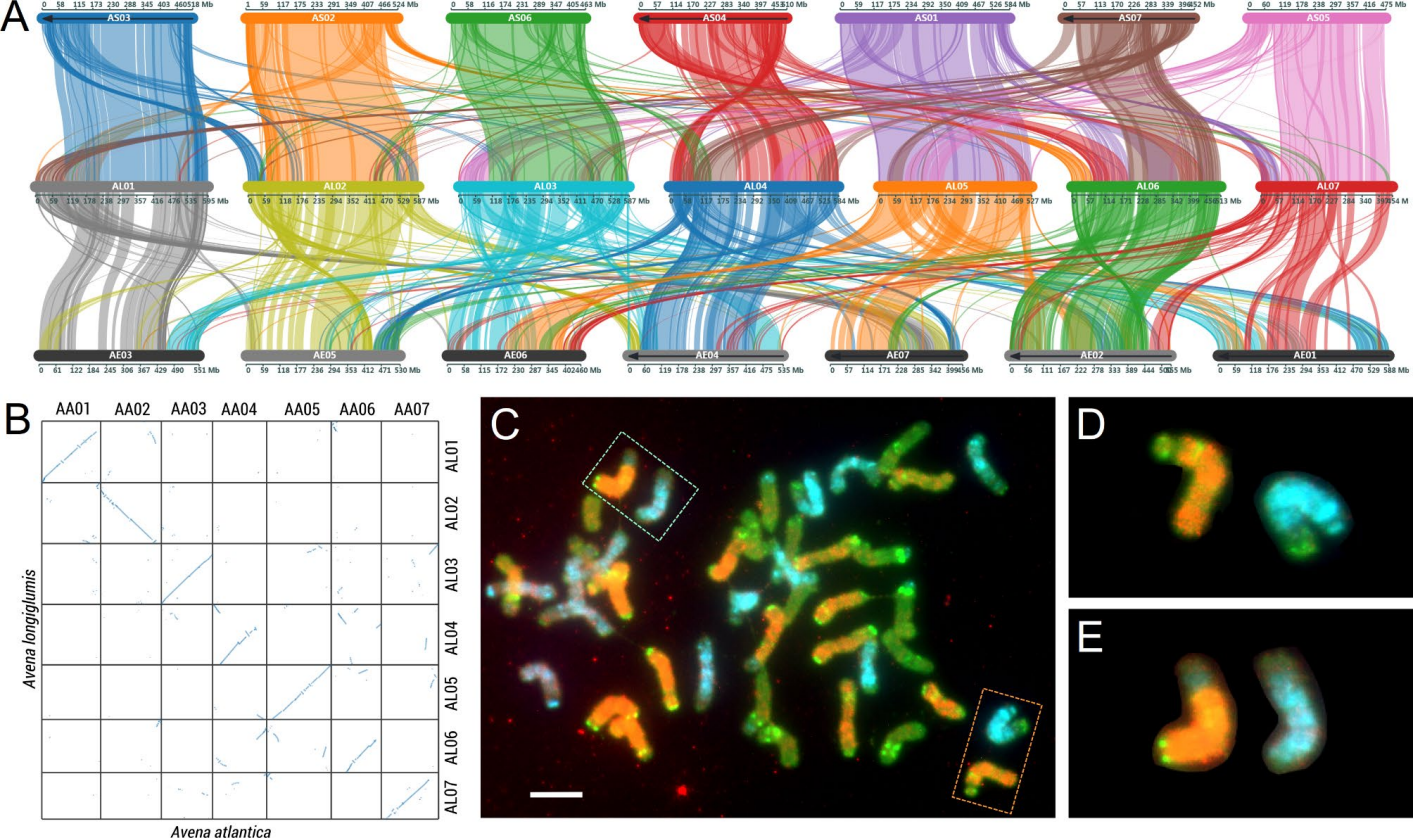
Syntenic relationship of *Avena strigosa* (AST), *A*. *longiglumis* (ALO), and *A*. *eriantha* (AER) genomes **A** Syntenic analysis of AST (top), ALO (middle), and AER (bottom). Subterminal regions are frequently involved in inter- and intra-specific evolutionary translocations. **B** Dotplot of AAT-ALO. **C–E** Fluorescent in situ hybridization (FISH) showing evolutionarily recent inter-genomic translocations between *Avena* genomes in the polyploid *A*. *sativa*. These are similar in extent to those identified by synteny analysis between *Avena* species. **C** FISH karyotype of *A*. *sativa*. Probes are AF226603_45bp (TET, pseudo-blue) for C genome, pAs120a (biotin, pseudo-red) for A genome, and Ab-T148 (digoxigenin, pseudo-green) for A/D genome. Probes were amplified from *A*. *longiglumis*. **D** Translocation shown by orange dotted square in Fig. 3**C**. **D** and **E** Translocation shown by green dotted square in Fig. 3**C**

To evaluate the intraspecific chromosome structure across the genus, we analyzed intragenomic synteny for AAT, ALO, AST, and AER. We found more than 21,000 pairs of collinear genes among ALO-AST, ALO-AER, and AER-AST species pairs (Additional file 2: Table S6). The greater number of rearrangements in AGK with respect to the AGK (Fig. 2) suggests it has the most derived karyotype in *Avena*, while A-genome species (ALO, AST, and AAT) are more primitive. Visualization of syntenic regions between ALO, AST and AER, shows large blocks of conservation between ALO and AST, with much more rearrangements with phylogenetically more distant AER (Fig. 3A). Between ALO and AER, AL01 was collinear with AE03, and AL06 with AE02. Notably, seven evolutionary inter-chromosomal translocations involved large distal domains between 10.64% and 37.24% of the chromosome length, not including centromeric translocations (Fig. 3, Additional file 2: Tables S7, S8, and S9 [50–56]). Figure 3B shows a dotplot comparison of AAT and ALO. Similar distal translocations to those between ALO, AER, and AST are evident, including an intrachromosomal translocation between the ends of chromosomes AA04 and AL05 (Fig. 3B, Additional file 1: Figure S5D).

These distal intragenomic evolutionary chromosomal rearrangements, now identified between diploid *Avena* species, are consistent with the distal nature and size of translocations identified using genome-specific repeat probes in polyploid *Avena* (Fig. 3C-E, see also Liu et al. [48]). In situ hybridization shows the relatively uniform dispersal of many genome-specific repetitive DNA sequences isolated from diploid *Avena* species, consistent with the uniform genomic expansion from the ancestral AGK species with a smaller genome. The hybridization pattern does not show bands of repeats interspersed with repeat-depleted genic regions (Fig. 3C and E). The translocations in hexaploid oat, involving different genomes, have occurred since hybridization and polyploidization but are not accompanied by homogenization of the repeats across the chromosomes, so translocations are revealed by the genome-specific probes. Distal translocations of similar nature and extent were found to occur during the evolution of the diploid species (Fig. 3), now accompanied by homogenization of the repeats so that they are only detected by the synteny analysis.

## Discussion

The presence of thousands of orthologous genes in multiple species has enabled remarkable comparisons of genomes and their evolution over long evolutionary distances [57–59]. Such studies have revealed the conservation of syntenic blocks and their rearrangements as mosaics. Among grasses, polyploidy has played a major part in the earliest (c. 110 Mya, with the ρ event) [4] and most recent (< 1 Mya, for example with tetraploid and hexaploid oat and wheat) events in Oryzoideae and Pooideae [11]. In the BOP clade, genomic expansion (Fig. 1) and chromosome reorganization with a number of well-defined events (Fig. 2) complementing wheat [19], indicate that gene duplication, gain, and loss have played a relatively small role. The 3.85 Gb chromosome-scale genome assemblies of *A*. *longiglumis* and other diploid *Avena* species have facilitated analyses of genomic expansion involving repetitive DNA amplification and homogenization (as discussed in Liu et al. [48] and Heslop-Harrison and Schwarzacher [60]). These data can be integrated into evolutionary models [6, 11] for comparison of the smaller genomes of rice and Brachypodium to the *Avena* genomes.

### Chromosomal block evolution and genomic expansion

The conservation of large syntenic blocks and orthologous relationships between seven ALO chromosomes, twelve chromosomes of OSA, and five of BDI, highlighted chromosomal block evolution with a well-defined number of fusion, translocation and nesting events, but few duplications or deletions (Figs. 1 and 2). Interestingly, certain chromosomes contained many rearrangements and smaller blocks of conserved synteny, while others remain more intact in the three species. The signature of the ρ-WGD event is evident (Additional file 1: Figure S5), and the lack of further large regions of synteny is indicative of a lack of major duplications in the *Avena* lineage. In contrast, the monocotyledons in the Musaceae [26, 61] exhibit evidence of three WGD events, while the PACMAD (sister to the BOP) clade contains a tetraploidization event in the maize lineage [62].

Our results revealed that the genomic expansion was uniform along chromosome arms, from telomeres to broader proximal regions around the centromeres, between *A*. *longiglumis* and rice (10.1-fold smaller) and Brachypodium (15.6-fold smaller) (Fig. 1). The lines of synteny in the dotplots comparing BDI and OSA with ALO (Fig. 1C, Additional file 1: Figure S1) are largely at the same slope (and straight), indicating equal expansion of all syntenic blocks throughout the larger genome. A few individual line segments were curved, indicating greater genomic expansion (spreading out the genes over a longer length) at one end than the other within a syntenic block. This is contrast with *Musa acuminata* and *Ensete glaucum* (two Musaceae species with similar genome sizes; Fig. 8B in Wang et al. [26]), where some lines of synteny were curved and many segments had different slopes. Our results do not support an alternative hypothesis about uneven genomic expansion. For example, we do not see large repeat-domains of integrating hot-spots interspersed between the syntenic blocks, except around the centromeres (supported by the relative uniformity of repeat distribution along chromosome arms in ALO, Additional file 1: Figures S3 and S4). In particular, blocks of tandemly repeated DNAs, often seen as heterochromatin, are not found in *Avena* except around the centromeres. This is in contrast to Triticeae species, which contain large terminal, centromeric, and intercalary blocks of tandem repeats [63]. The expansion of the *Avena* genome with respect to BDI and OSA is largely, although not entirely, accounted for the annotated repetitive element expansion. Specially, 87% of ALO was represented by annotated repeats, compared to 36% in BDI and 48% in OSA, giving repeat-masked genome sizes of 513, 167 and 203 Mb respectively (Additional file 2: Table S8A and B).

In other Pooideae species, a collinearity analysis between *Lolium perenne* (2,550 Mb) and barley (4,830 Mb) genomes shows even expansion throughout the chromosomes, and no chromosomal segments with markedly stronger sequence expansion or contraction [64]. In contrast, synteny between the three hexaploid wheat (*Triticum aestivum*, 2*n* = 6*x* = 42) genomes, derived from diploid species *Triticum urartu* (A genome), an *Aegilops speltoides*-related species (B), and *Aegilops tauschii* (D), have uncovered that the smaller D genome, compared to the B genome, shows notable lines of synteny connecting homoeologous genes with gaps or discontinuities (centre of the Circos plot Fig. 1 in El Baidouri et al. [19]), which are not seen between the *Avena* species (Fig. 3A), nor between ALO and BDI or OSA (Fig. 1A, Additional file 1: Figures S2). It is likely that the gaps in the B genome are composed of gene-poor heterochromatic repetitive elements, well-known in the *Aegilops* ancestral species but not seen outside the centromeric region in *Avena* [48].

Massive oligonucleotide pools, synthesizing tens of thousands of synthetic labeled probes, are proving valuable for chromosome evolution studies [65, 66]. Given the extensive gene homology between the BOP grasses shown here, these may enable the design of synthetic chromosome oligonucleotide pools for in situ hybridization to identify syntenic chromosomal blocks and their rearrangements. The use of multiple baits allows isolation of orthologous (and sometimes paralogous) genes from multiple species in the Angiosperm353 projects [67–69] for phylogenetic studies, and it would be exciting if a related probe pool technology could be used to track chromosomal reorganization.

### Chromosome evolution during oat diploid speciation

Despite their relatively close relationship, separating between 2 and 10 Mya, we identified substantial rearrangements of syntenic blocks of genes between the four diploid *Avena* species studied here (Figs. 2 and 3). The more diverged AER (designated as C-genome) exhibited more rearrangements than the A-genome species AAT, ALO, and AST. Notably, multiple translocations involving distal regions of chromosome arms were clear from AST to ALO (Fig. 3A, Additional file 1: Figure S1B); and from ALO to AAT (Fig. 3B). The three *Avena* A genomes, with frequent terminal segment translocations, contrast with the Triticeae [19] that show near end-to-end synteny, with no distal arm translocations between 2*x* wheat ancestors (except chromosome 5 A; compare centre of Fig. 1 in El Baidouri et al. [19] with Fig. 3A here). The wheat diploid ancestor phylogenetically separated over an approximately similar period to the *Avena* species. Thus, we postulate that chromosomal rearrangements have been more active or perhaps more stably in *Avena* compared to wheats (*Triticum*), although both tribes have a conserved chromosome number of *x* = 7.

During and following speciation, many of the repetitive elements identified from *Avena* diploid species have become species-specific [48] and, as in Brassicaceae [12], are phylogenetically informative. The sequences have been replaced, lost, amplified, and homogenized along all chromosome arms [48], with little change in *Avena* genome size (cf., genomic expansion in Fig. 1; and *Avena* species in Fig. 3). While broad pericentromeric regions are reservoirs for accumulation of a medley of TEs [26, 60, 70].

In situ hybridization using genome (species)-specific repeat probes shows that in the hexaploid *A. sativa* (Fig. 3C–E), many chromosomes contain intergenomic translocations between chromosomes of diploid genomes [71, 72], involving the terminal 10.64–37.24% of chromosome arms (Additional file 2: Table S7). Our analysis of conserved gene synteny (Fig. 3A and B) revealed that the four diploid *Avena* species (ALO, AST, AAT, and AER) contain multiple terminal translocations between chromosomes. Notably, the terminal rearrangements involved more than just repetitive DNAs, as is the case in maize (The P53 knob) [73] or rye (pSc250 tandem repeat) [63], and include many genes in the synteny [74, 75]. The hexaploid result shows that distal translocation events in *Avena* continue to occur post-polyploidization, between chromosomes of different species origin (Fig. 3C–E).

Both genomic expansion and chromosomal rearrangement have occurred during evolution of *Avena* from a proposed AGK similar to rice, without further rounds of polyploidy. Chromosomal structural variation is extensive, and may restrict hybridization and lead to reproductive isolation. While a key feature of speciation, this phenomenon restricts crossing in breeding programms to exploit wider germplasm pools. Chromosome structural variation is increasingly recognized as a factor controlling complex traits in livestock [76] and crop plants [77], and must be discerned as a part of the pangenome [26, 78]. The 10-fold to 15-fold genomic expansion involving relatively uniform interspersion of genes with repetitive DNAs throughout chromosome arms, along with changes in the size of gene-depleted broad centromeric regions, may also contribute to modulation of gene expression, and perhaps reproductive isolation, although meiotic pairing can compensate for substantial genome-size differences [79].

Insight into the extent and nature of chromosomal rearrangements and genomic expansion in the pangenome is critical for identifying the processes of evolution and speciation. Beyond the level of gene sequences, this information can inform studies on biodiversity, and contribute to the exploitation of diversity present in the common gene pool across grasses through precision breeding. Pangenomic resources will allow us to increase power in genome editing and synthetic biology [80], reduce costs by saving resources required for extensive phenotypic selections [81], speed up the process of genetic improvement [82], and realize the genetic gains per unit time with high precision [83]. Therefore, the knowledge gained in the genomic expansion and reorganization of the BOP clade can be rapidly transferred to exploit biodiversity and widen gene pools available to the genomic-assisted breeding programs for future crops [84, 85].

### Electronic supplementary material

Below is the link to the electronic supplementary material.

Supplementary Material 1

## Data Availability

The raw sequencing reads of Nanopore, Hi-C and Illumina sequencing data that were used for the genome assembly have been deposited in the NCBI Sequence Read Archive with accession number SRR19279519-SRR19279520 (Nanopore), SRR19279522-SRR19279531 (Nanopore), SRR19279511-SRR19279517 (Hi-C), SRR19279521, SRR19279532-SRR19279533 (Hi-C), and SRR19279518 (Survey data) under BioProject accession number PRJNA838431. The assembly data have been deposited in the NCBI under the BioProject ID PRJNA956334. The chromosomal assembly and annotation are also available on Figshare with the identifier 10.6084/m9.figshare.19130429.v2.

## Abbreviations

AAT: *Avena atlantica*
AER: *A. eriantha*
AGK: ancestral grass karyotype
ALO: *A. longiglumis*
AST: *A. strigosa*
BDI: *Brachypodium distachyon*
BOP: Bambusoideae, Oryzoideae, and Pooideae
DAPI: *4’, 6-diamidino-2-phenylindole*
EDTA: ethylenediamine-tetraacetic acid
FISH: Fluorescence in situ hybridization
FITC: fluorescein isothiocyanate
Hi-C: high-throughput chromatin conformation capture
Mb: Megabases
Mya: million years ago
OSA: *Oryza sativa*
NCBI: National Center for Biotechnology Information
PACMAD: Panicoideae, Arundinoideae, Chloridoideae, Micrairoideae, Aristidoideae, and Danthonioideae
SDS: sodium dodecyl sulphate
SSC: saline sodium citrate
WGD: whole-genome duplication
