# Supplementary material for: Genome-wide expansion and reorganization during grass evolution: from 30 Mb chromosomes in rice and Brachypodium to 550 Mb in Avena
Source: BMC Plant Biol. 2023 Dec 8;23:627. doi: 10.1186/s12870-023-04644-7 (PMC10704644; doi:10.1186/s12870-023-04644-7)
Supplement: Supplementary file 1 — Supplementary Material 1 [file 12870_2023_4644_MOESM1_ESM.docx]

**Qing Liu et al. Genome-wide chromosomal expansion in grasses. Additional file 1: Figures S1–S5.**

**Additional file 1: Figure S1.** Syntenic relationships between the AAT, AST, ALO, AER, BDI, and OSA genomes showing homologous regions in SynVisio plots or dotplots. **Figure S2.** SynVisio plots between *Oryza sativa* and *A. longiglumis* and *Brachypodium distachyon* genomes showing major syntenic regions. **Figure S3.** Gene density (number of genes in 1 Mb-sized window) on seven chromosomes of *A. longiglumis*. **Figure S4.** The locations of the centromeric retrotransposon *Cereba* (KM948610) on seven chromosomes of *A. longiglumis*. **Figure S5.** Ancient *ρ* (rho) whole genome duplications (WGD) in *Avena*.


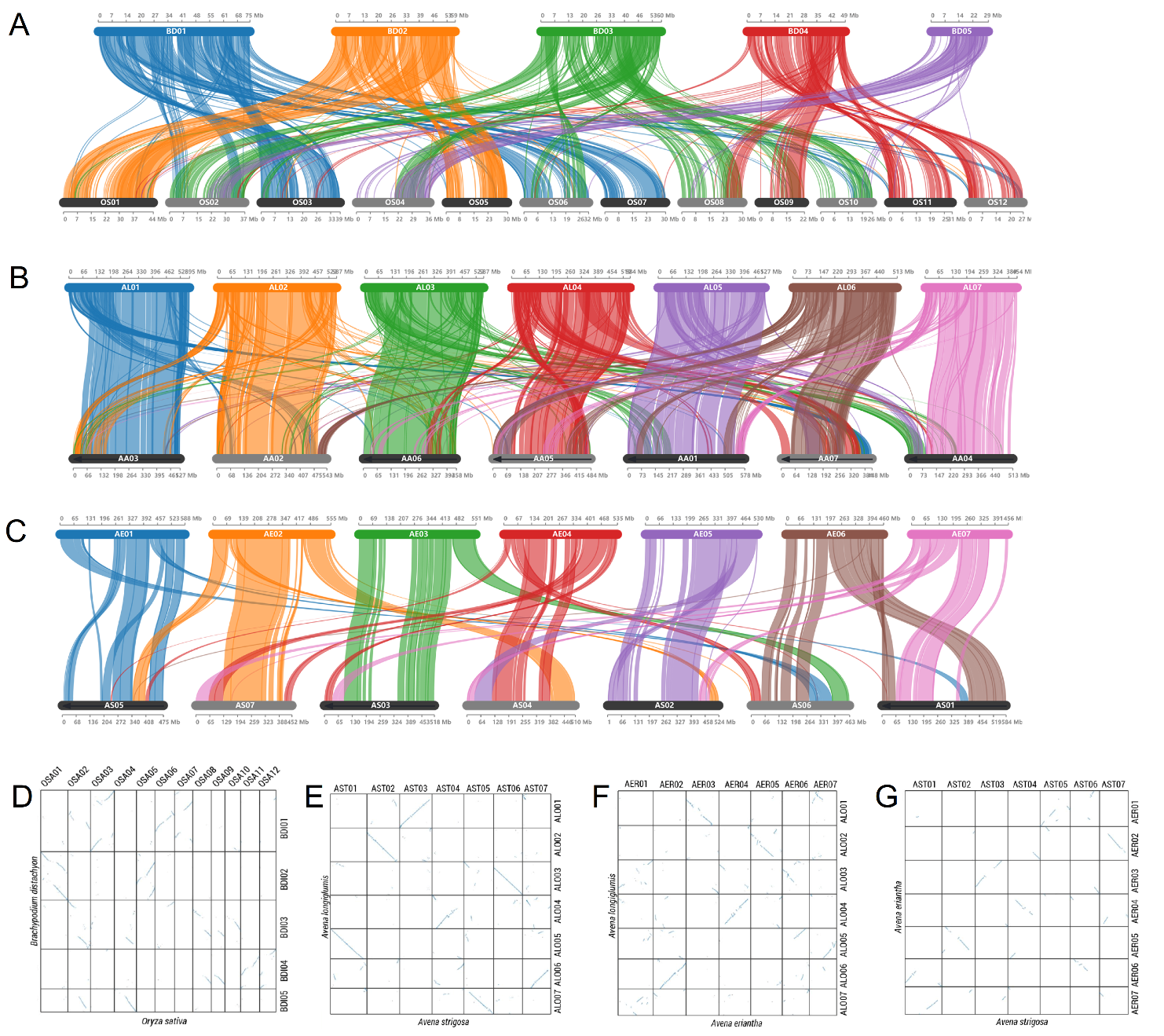
 Figure S1. Syntenic relationships between the AAT, AST, ALO, AER, BDI, and OSA genomes showing homologous regions in SynVisio plots or dotplots. A Syntenic analysis of BDI and OSA. B Syntenic analysis of ALO and AAT. C Syntenic analysis of AER and AST. D Dotplot of OSA-BDI genomes. E Dotplot of AST-ALO genomes. F Dotplot of AER-ALO genomes. G Dotplot of AST-AER genomes.


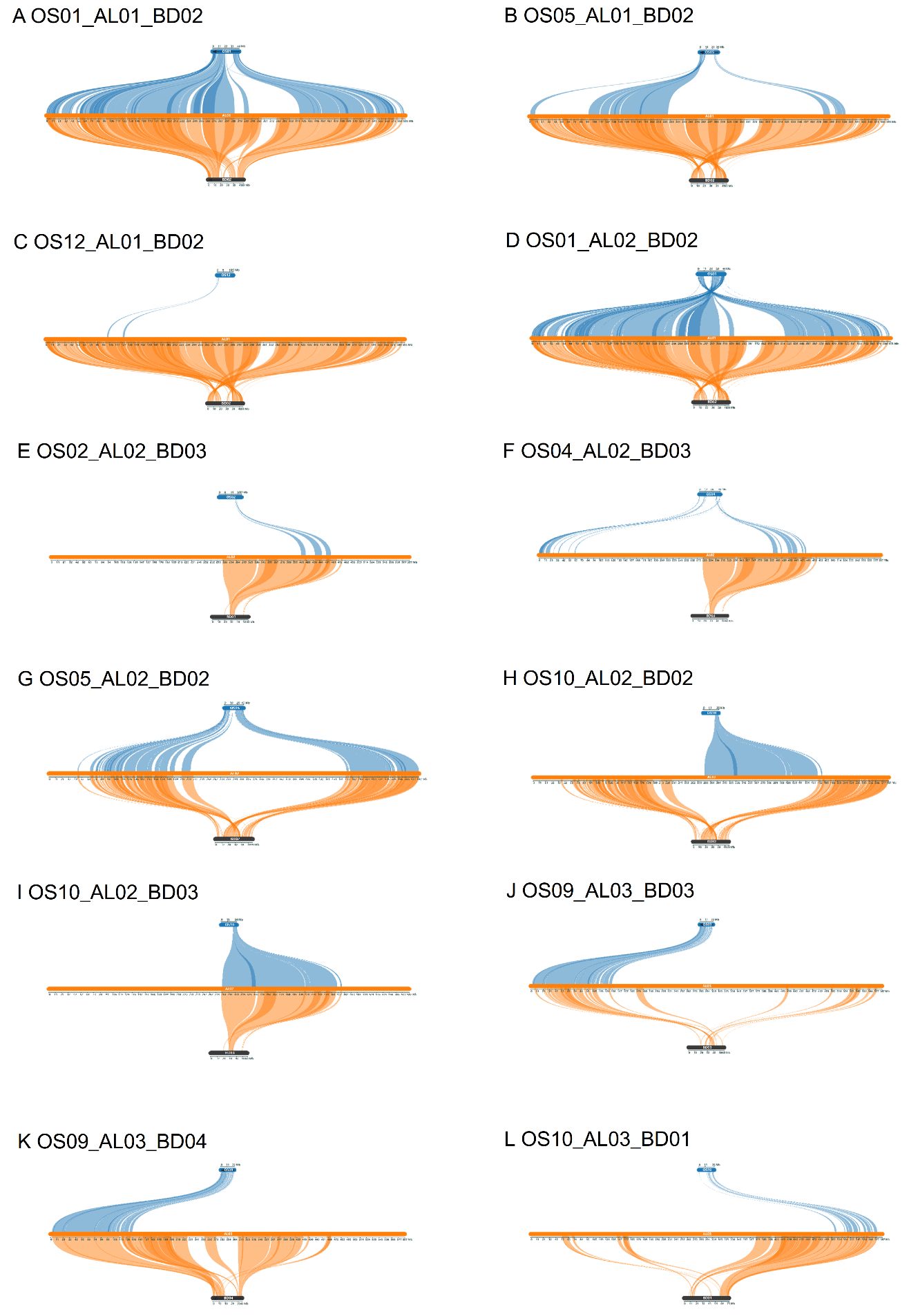


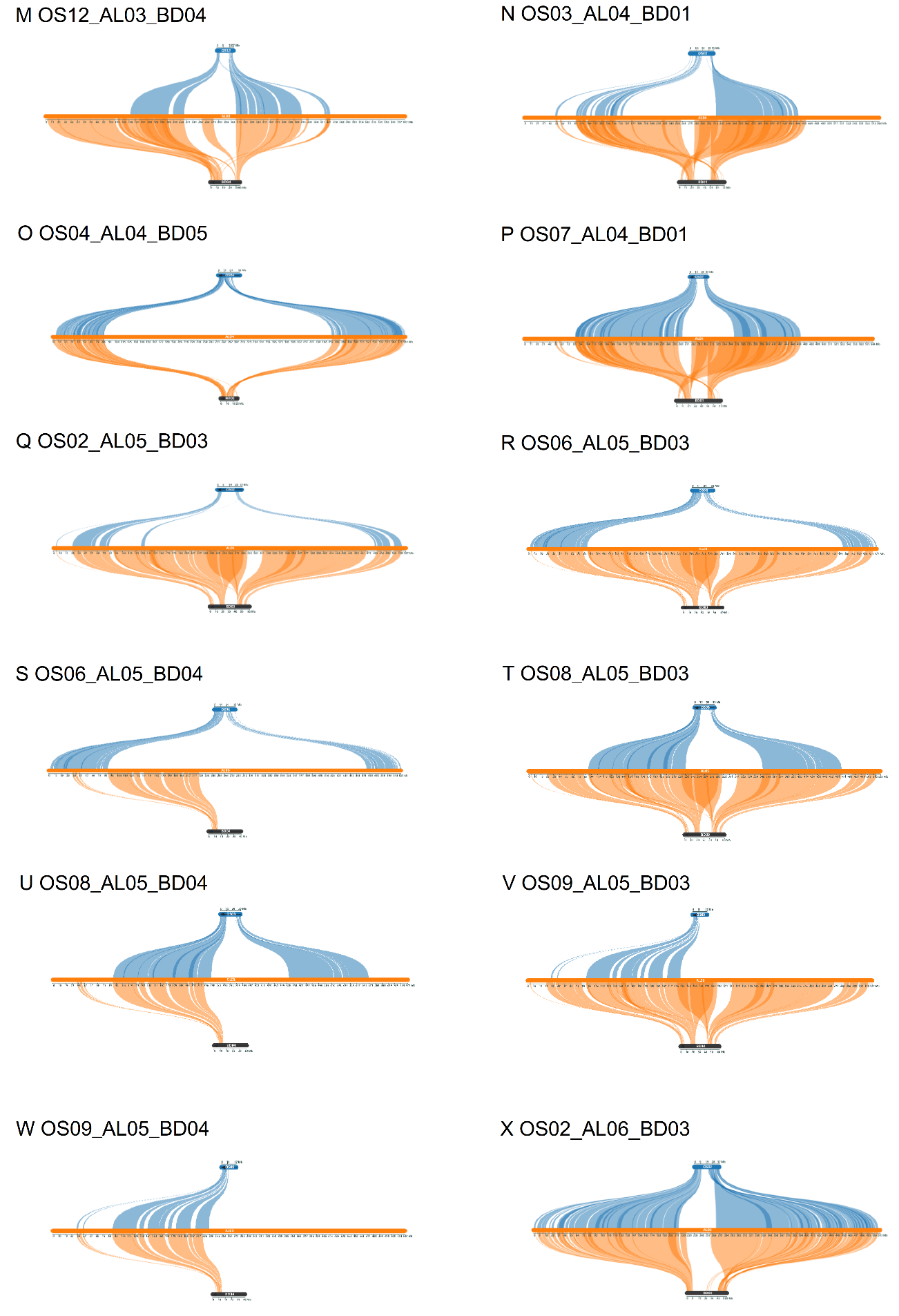

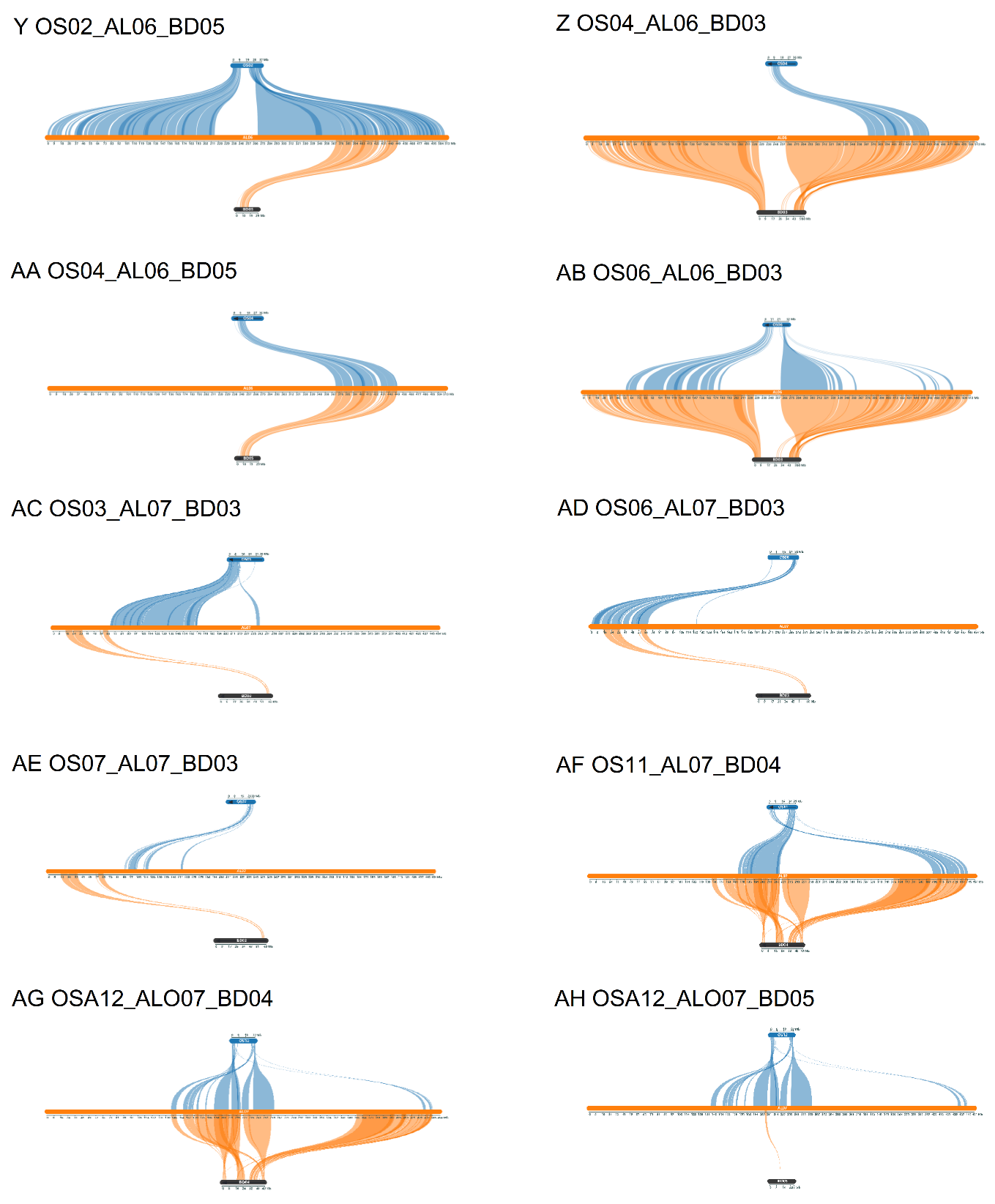


Figure S2. SynVisio plots between *Oryza sativa* and *A. longiglumis* and *Brachypodium distachyon* genomes showing major syntenic regions. A OS01-AL01-BD02. B OS05-AL01-BD02. C OS12-AL01-BD02. D OS01-AL02-BD02. E OS02-AL02-BD03. F OS04-AL02-BD03. G OS05-AL02-BD02. H OS10-AL02-BD02. I OS10-AL02-BD03. J OS09-AL03-BD03. K OS09-AL03-BD04. L OS10-AL03-BD01. M OS12-AL03-BD04. N OS03-AL04-BD01. O OS04-AL04-BD05. P OS07-AL04-BD01. Q OS02-AL05-BD03. R OS06-AL05-BD03. S OS06-AL05-BD04. T OS08-AL05-BD03. U OS08-AL05-BD04. V OS09-AL05-BD03. W OS09-AL05-BD04. X OS02-AL06-BD03. Y OS02-AL06-BD05. Z OS04-AL06-BD03. AA OS04-AL06-BD05. AB OS06-AL06-BD03. AC OS03-AL07-BD03. AD OS06-AL07-BD03. AE OS07-AL07-BD03. AF OS11-AL07-BD04. AG OS12-AL07-BD04. AH OS12-AL07-BD05.


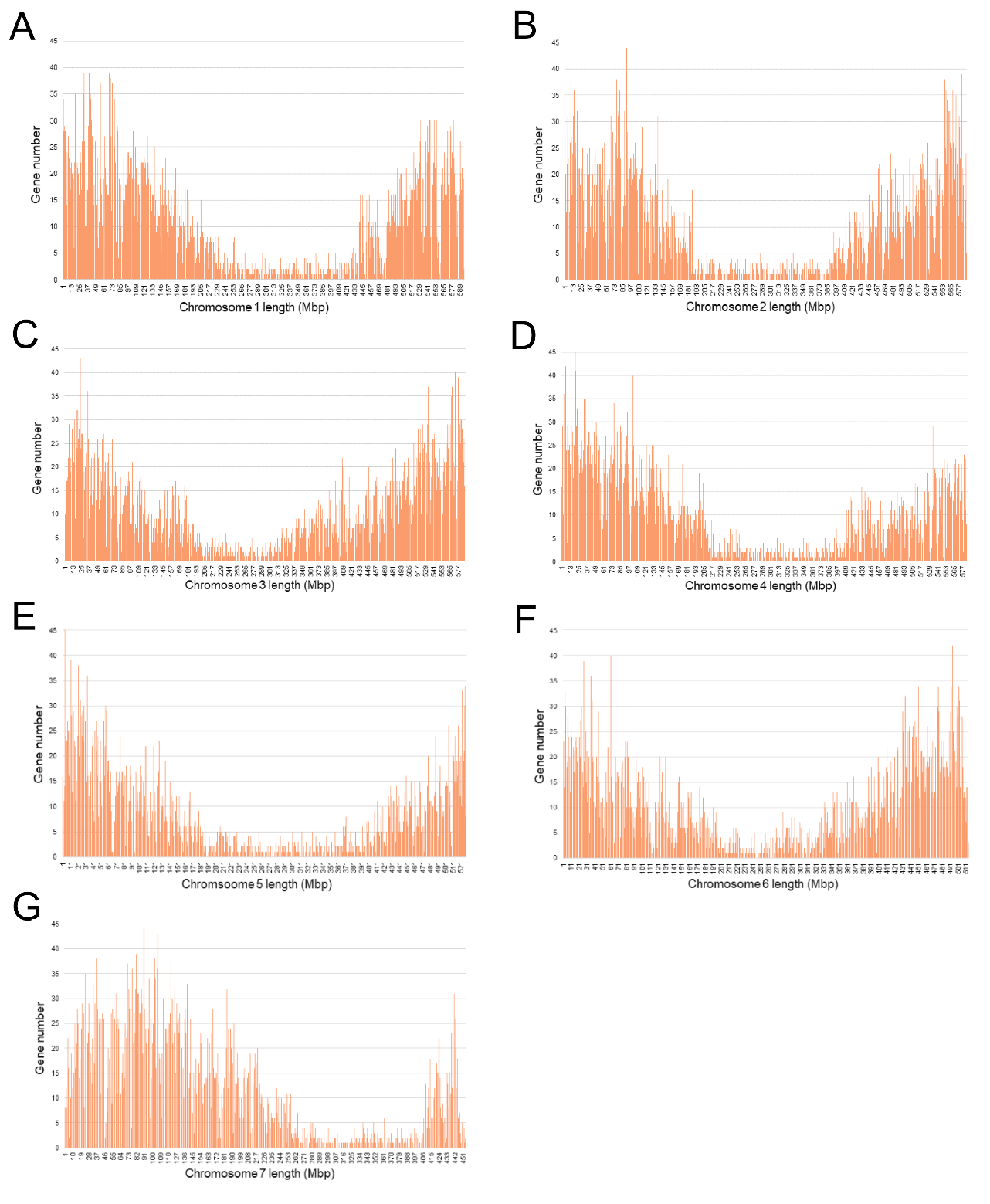


Figure S3. Gene density (number of genes in 1 Mb-sized window) on seven chromosomes of *A. longiglumis*. A AL01 histogram. B AL02 histogram. C AL03 histogram. D AL04 histogram. E AL05 histogram. F AL06 histogram. G AL07 histogram.


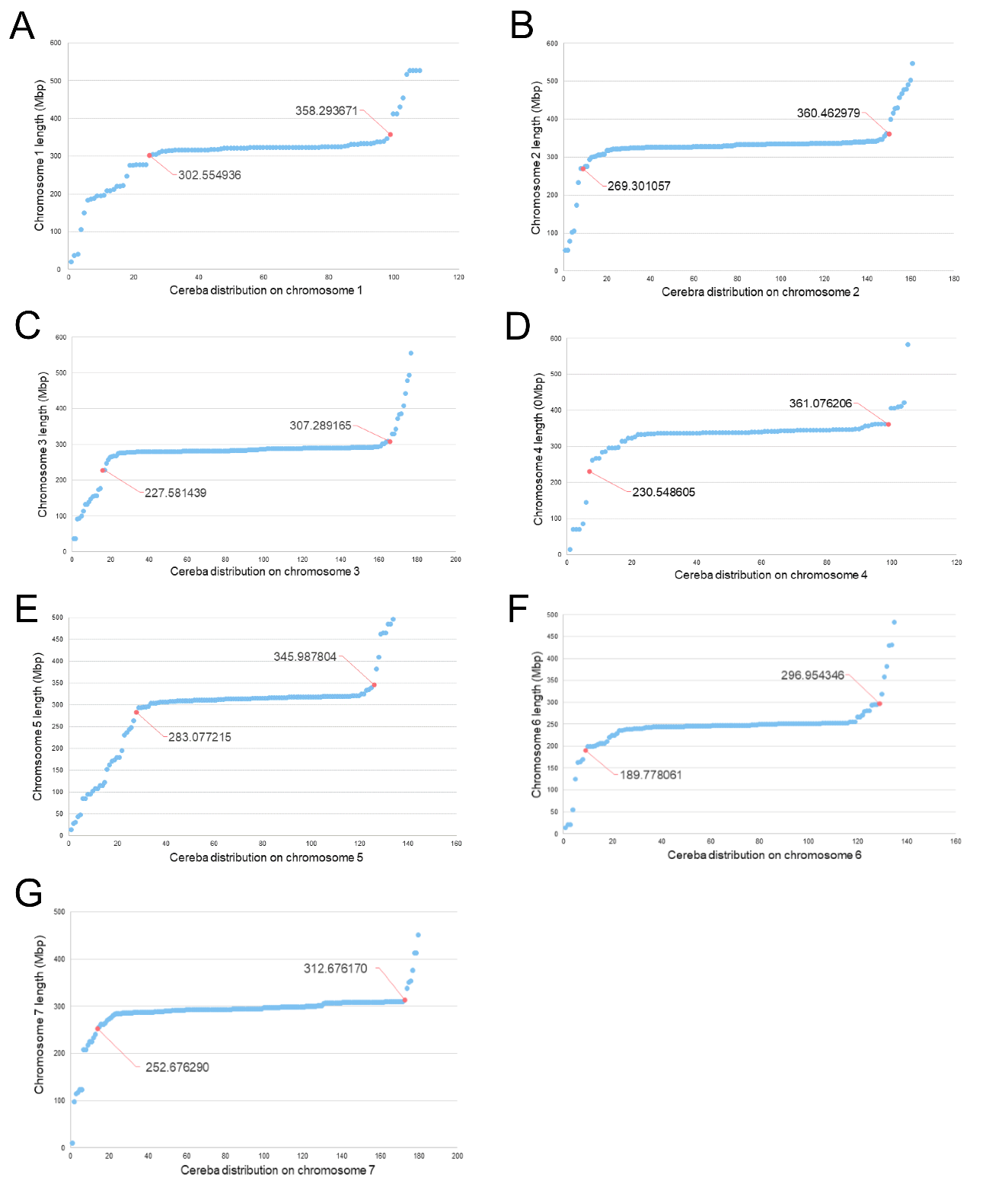
 Figure S4. The locations of the centromeric retrotransposon *Cereba* (KM948610[37]) on seven chromosomes of *A. longiglumis*. Regions spanning the centromere denoted by red dots and coordinates. A Relatively continuous distribution of *Cereba* from 302,554,936 bp to 358,293,671 bp on chromosome 1 of *A*. *longiglumis* (AL01). B Relatively continuous distribution of *Cereba* from 269,301,057 bp to 360,462,979 bp on AL02. C Relatively continuous distribution of *Cereba* from 227,581,439 bp to 307,289,165 bp on AL03. D Relatively continuous distribution of *Cereba* from 230,548,605 bp to 361,076,206 bp on AL04. E Relatively continuous distribution of *Cereba* from 283,077,215 bp to 345,987,804 bp on AL05. F Relatively continuous distribution of *Cereba* from 189,778,061 bp to 296,954,346 bp on AL06. G Relatively continuous distribution of *Cereba* from 252,676,290 bp to 312,676,170 bp on AL07.


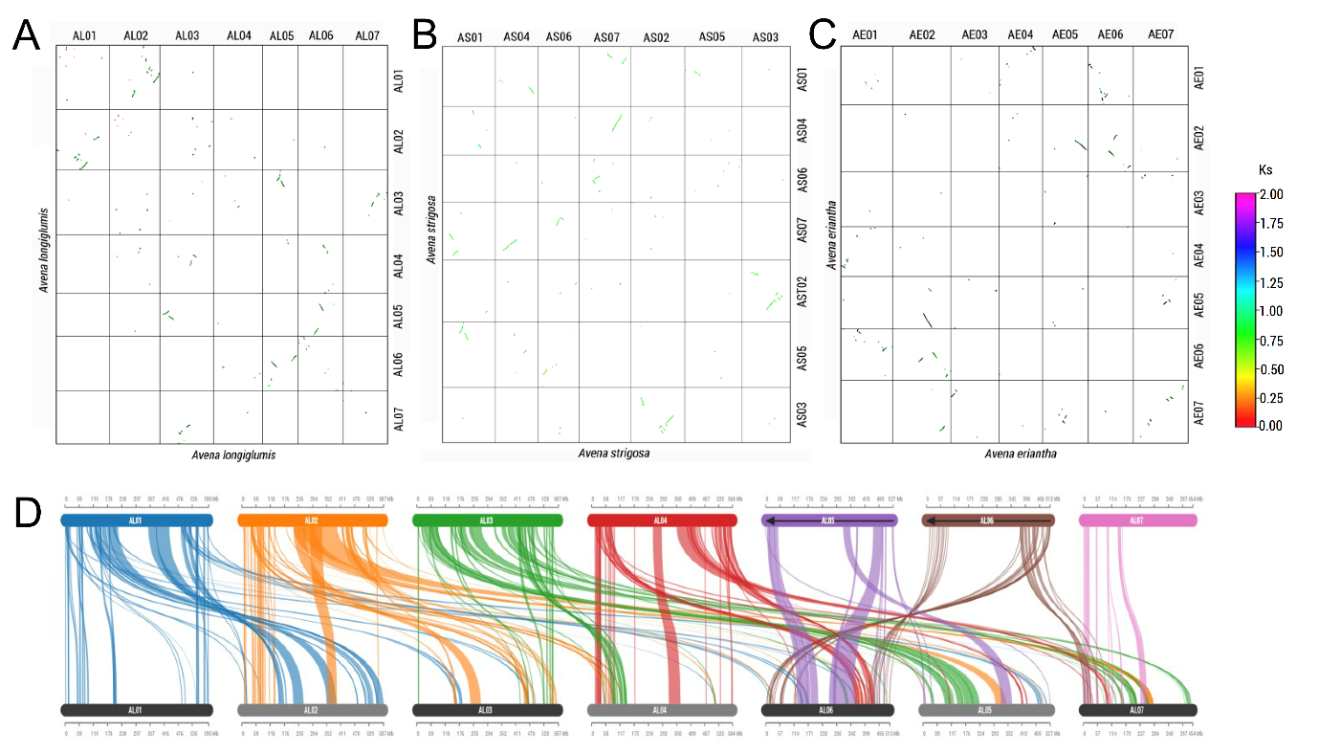
 Figure S5. Ancient ρ (rho) whole genome duplications (WGD) in *Avena*. A*–*C Self-dotplots (not showing the principal diagonals) of ALO (A), AST (B), and AER (C). D SynVisio plots (with self-identity masked) of *A. longiglumis* genome showing signature of ancient ρ duplication.

# Qing Liu et al. Genome-wide chromosomal expansion in grasses. Additional file 2: Tables S1–S9.

# Table S1. Deposited data pertaining to the sequencing, assembly, and annotation of *A. longiglumis* (ALO) used in the study.

| Sequencing and assembly | Deposited data |
| --- | --- |
| Sequencing data for ALO | National Center for Biotechnology Information (NCBI) database under BioProject code PRJNA838431 [Genome survey data (SRR19279518), ONT data (SRR19279519-SRR19279520 and SRR19279522-SRR19279531), and Hi-C data (SRR19279511-SRR19279517, SRR19279521 and SRR19279532-SRR19279533)] |
| Genome assembly and annotation for ALO | NCBI SRA BioProject code PRJNA956334 and annotation file deposited in FigShare repository https://doi.org/10.6084/m9.figshare.19130429.v2. |

# Table S2. Summary of genome assembly and annotation of *A. longiglumis* (ALO).

## **A** Statistics of the ALO genome assembly.

| Genome features | Measures of index |
| --- | --- |
| Genome survey |  |
| Illumina sequences | 267.54 Gbp (67.55×) |
| Genome survey contigs | 4,040,426 |
| *K*-mer | 17 |
| *K*-mer number | 208,165 |
| *K*-mer depth | 49.80 × |
| Estimated genome size | 3,965,670,000 bp |
| Heterozygous ratio (%) | 0.48 |
| Repeat (%) | 82.08 |
| Genome assembly |  |
| Nanopore sequences | 252.78 Gbp (63.82×) |
| Nanopore contig number | 2,379 |
| Nanopore contig number correction | 2,381 |
| Assembled genome size | 3,960,768,570 bp |
| Assembly % of genome | 99.88% |
| Longest contigs | 99,445,397 bp |
| Contig N50 length | 12,682,464 bp |
| Contig N90 length | 662,566 bp |
| Contig L50 count | 74 |
| Contig L90 count | 806 |
| Number of scaffolds | 414 |
| Total size of scaffolds | 3,960,965,270 bp |
| Scaffold N50 length | 527,343,613 bp |
| Scaffold N90 length | 453,691,697 bp |
| Scaffold N50 count | 5 |
| Scaffold N90 count | 9 |
| Longest scaffold | 594,546,470 bp |
| GC content | 44.67% |
| Hi-C auxiliary assembly |  |
| Library data | 435.64 Gb |
| Raw Paired-end reads | 2,904,215,238 |
| Clean Paired-end reads | 2,870,596,248 |
| Clean bases | 420,725,014,170 bp |
| Clean Q30 bases | 394,303,483,280 (99.55×) |
| Clean paired-end read rate | 98.84% |
| Clean Q30 base rate | 93.72% |
| Hi-C contig number | 2381 |
| Longest contig length | 99,445,397 |
| Contig N50 length  Contig N90 length  Total size of contigs  Gap length  Total size of scaffolds | 12,682,464  661,603  3,960,768,570  196,700  3,960,965,270 |
| Total length of pseudo-chromosomes (% assembly) | 3,847,578,604 (97.14%) |
| Hi-C scaffold number | 414 |
| Longest scaffold length | 594,546,470 |
| Scaffold N50 length | 527,343,613 |
| Scaffold N90 length | 6,968,329 |
| Gene models |  |
| Number of predicted genes | 40,845 |
| Number of annotated genes | 39,491 |
| Number of HC genes (Proportion of genes) | 33,271 (81.46%) |
| Number of LC genes (Proportion of genes) | 7,574 (18.54%) |
| Mean gene density | 10 / Mbp |
| Total length of genes (bp) | 133,632,290 |
| Number of genes | 40,845 |
| Mean gene length (bp) | 3,271.69 |
| Total length of exons (bp) | 50,244,372 |
| Number of exons | 187,479 |
| Mean exon length (bp) | 268 |
| Mean exon number | 4.59 |
| Total length of HC genes (bp) | 115,042,270 |
| Total length of LC genes (bp) | 18,590,020 |
| Number of HC genes (Proportion of genes) | 33,271 (81.46%) |
| Number of LC genes (Proportion of genes) | 7,574 (18.54%) |
| Mean HC gene length (bp) | 3457.73 |
| Mean LC gene length (bp) | 2454.45 |
| Number of annotated genes | 39,491 |
| Number of mRNA | 15,446 |
| Mean exon number per HC gene | 5.02 |
| Mean exon length of HC genes | 1,306 bp |
| Mean intron length of HC genes | 2,152 bp |
| Nonprotein coding genes/elements |  |
| Number of miRNA genes | 15,446 |
| Mean length of miRNA genes | 136.29 bp |
| miRNA genes share in genome | 0.0532% |
| Number of rRNA genes | 257 |
| Mean length of rRNA genes | 901.50 bp |
| rRNA genes share in genome | 0.0058% |
| Number of tRNA genes | 1,402 |
| Mean length of tRNA genes | 75.62 bp |
| tRNA gene share in genome | 0.0027% |
| Number of snRNA genes | 607 |
| Mean length of snRNA genes | 101.92 bp |
| snRNA genes share in genome | 0.0016% |
| Total repetitive DNAs | 3,479,237,283 bp |
| Repeat region % of assembly | 87.04% |
| Total transposable elements | 3,411,928,666 bp |
| Percentage of transposable element in genome | 86.14% |

## B Summary of Oxford Nanopore Technologies assembly metrics.

|  | Contigs  (after polish) | Scaffold  (Nanodrop + Hi-C) |
| --- | --- | --- |
| Total length | 3,960,768,570 bp | 3,960,965,270 bp |
| Number of contigs | 2,379 | 414 |
| Maximum contig length | 99,445,397 bp | 594,546,470 bp |
| N50 | 12,682,464 bp | 527,343,613 bp |
| N60 | 7,675,489 bp | 513,337,126 bp |
| N70 | 3,127,915 bp | 453,691,697 bp |
| N80 | 1,419,177 bp | 9,709,002 bp |
| N90 | 662,566 bp | 6,968,329 bp |

# Table S3. Gene family statistics of ALO and other analyzed Pooideae species.

| Species (Abbreviation) | Genome size (Mbp) | Gene number | Genes in families | Unclustered genes | Gene family number | Unique gene family number | Average gene number per family |
| --- | --- | --- | --- | --- | --- | --- | --- |
| *A. atlantica* (AAT) | 3,690 | 49,542 | 44,363 | 5,179 | 22,813 | 845 | 1.94 |
| *A. eriantha* (AER) | 3,780 | 47,361 | 42,182 | 5,179 | 21,750 | 818 | 1.94 |
| *A. longiglumis* (ALO) | 3,961 | 40,845 | 39,288 | 1,557 | 19,954 | 259 | 1.97 |
| *A. strigosa* (AST) | 4,100 | 39,885 | 37,574 | 2,311 | 21,620 | 355 | 1.74 |
| *Brachypodium distachyon* (BDI) | 271 | 32,439 | 28,251 | 4,188 | 18,743 | 300 | 1.51 |
| *Oryza sativa* (OSA) | 389 | 42,189 | 35,655 | 6,534 | 19,870 | 1,351 | 1.79 |

# Table S4. Gene family categories in genomes of ALO and other analyzed Pooideae species.

| Species (Abbreviation) | Single-copy orthologs | Multiple-copy orthologs | Unique orthologs | Other orthologs | Unclustered genes |
| --- | --- | --- | --- | --- | --- |
| *A. atlantica* (AAT) | 1,880 | 12,995 | 2,973 | 26,515 | 5,179 |
| *A. eriantha* (AER) | 1,880 | 12,818 | 2,541 | 24,943 | 5,179 |
| *A. longiglumis* (ALO) | 1,880 | 15,329 | 869 | 21,210 | 1,557 |
| *A. strigosa* (AST) | 1,880 | 12,570 | 1,012 | 22,112 | 2,311 |
| *Brachypodium distachyon* (BDI) | 1,880 | 11,716 | 840 | 13,815 | 4,188 |
| *Oryza sativa* (OSA) | 1,880 | 12,304 | 5,268 | 16,203 | 6,534 |

#

# Table S5. Size and centromere localization of the ALO pseudomolecules.

## A Centromere localization results from high abundance areas of repeat sequences on chromosome dotplots.

| Chromosomes | Size (bp) | Suspected centromere | Start (bp) | End (bp) | Median (bp) |
| --- | --- | --- | --- | --- | --- |
| ALO01 | 594,546,470 | centormere1 | 315,061,249 | 324,147,201 | 319,604,225 |
| ALO02 | 587,543,788 | centormere1 | 323,004,417 | 335,723,521 | 329,363,969 |
| ALO03 | 587,190,583 | centormere1 | 280,098,446 | 290,268,161 | 285,183,304 |
| ALO04 | 583,925,327 | centormere1 | 336,345,261 | 345,614,337 | 340,979,799 |
| ALO05 | 527,343,613 | centormere1 | 305,957,889 | 318,402,561 | 312,180,225 |
| ALO06 | 513,337,126 | centormere1 | 245,726,549 | 250,604,796 | 248,165,673 |
| ALO06 | 513,337,126 | centormere2 | 252,557,313 | 253,143,041 | 252,850,177 |
| ALO07 | 453,691,697 | centormere1 | 288,095,587 | 297,553,921 | 292,824,754 |

## B Centromere localization result from mapping of centromeric retrotransposon Cereba (KM948610) (61) mapping result on ALO chromosomes.

| Chromosomes | Size (bp) | Start (bp) | End (bp) | Median (bp) |
| --- | --- | --- | --- | --- |
| ALO01 | 594,546,470 | 302,554,936 | 358,293,671 | 330,424,304 |
| ALO02 | 587,543,788 | 269,301,057 | 360,462,979 | 314,882,018 |
| ALO03 | 587,190583 | 227,581,439 | 307,289,165 | 267,435,302 |
| ALO04 | 583,925,327 | 230,548,605 | 361,076,206 | 295,812,406 |
| ALO05 | 527,343,613 | 283,077,215 | 345,987,804 | 314,532,510 |
| ALO06 | 513,337,126 | 189,778,061 | 296,954,346 | 243,366,204 |
| ALO07 | 453,691,697 | 252,676,290 | 312,676,170 | 282,676,230 |

## C Centromere localization results from conserved regions between A. longiglumis and Oryza sativa genomes using SynVisio result.

| Chromosomes | Start alignment no. | End alignment no. | Start (bp) | End (bp) | Median (bp) |
| --- | --- | --- | --- | --- | --- |
| ALO01 | 73-139 | 41-3 | 218,336,850 | 456,530,974 | 337,433,912 |
| ALO02 | 148-8 | 186-8 | 218,434,180 | 372,761,714 | 295,597,947 |
| ALO03 | 303-3 | 297-67 | 215,567,169 | 380,159,203 | 297,863,186 |
| ALO04 | 417-428 | 341-2 | 209,204,572 | 325,956,657 | 267,580,615 |
| ALO05 | 506-1 | 485-14 | 214,260,721 | 370,096,114 | 292,178,418 |
| ALO06 | 564-6 | 585-11 | 213,285,659 | 282,988,269 | 248,136,964 |
| ALO07 | 687-39 | 663-0 | 262,540,264 | 403,854,365 | 333,197,315 |

# Table S6. The gene pair statistics of SynVisio results between grass species and between post-ρ ancestral grass karyotype (AGK) and grass species.

| Sample 1 | Sample 2 | Number of genome blocks | Number of gene pairs | Number of gene pairs between samples 1_1 | Number of gene pairs between samples 1_2 | Number of gene pairs between samples 2_2 |
| --- | --- | --- | --- | --- | --- | --- |
| Grass species OSA_ALO_BDI | | | |  |  |  |
| ALO | OSA | 697 | 19,178 | 0 | 19,178 | 0 |
| ALO | BDI | 778 | 22,004 | 0 | 22,004 | 0 |
| BDI | OSA | 582 | 20,460 | 0 | 20,460 | 0 |
| Grass species AST-ALO-AER | | | | | | |
| ALO | AST | 670 | 29030 | 0 | 29,030 | 0 |
| ALO | AER | 824 | 27,116 | 0 | 27,116 | 0 |
| AER | AST | 172 | 21,536 | 0 | 21,536 | 0 |
| AGK and grass species | | | | | | |
| AGK | AAT | 505 | 14,400 | 1,013 | 11,579 | 1,808 |
| AGK | AER | 438 | 14,402 | 1,016 | 11,541 | 1,845 |
| AGK | ALO | 732 | 16,670 | 990 | 12,362 | 3,,318 |
| AGK | AST | 380 | 12,533 | 1,004 | 11,790 | 1,957 |
| AGK | ATA | 490 | 12,995 | 1,015 | 10,473 | 1,507 |
| AGK | BDI | 595 | 15,497 | 1,035 | 12,539 | 1,923 |
| AGK | OSA | 487 | 18,690 | 2,007 | 13,797 | 2,886 |

#

# Table S7. Terminal translocation size and percentage in ALO chromosomes determined from SynVisio results of AST-ALO-AER genomes (Figure 3A).

| Chromosome | Length (bp) | Centromere middle position | Left arm length (bp) | Right arm length (bp) | Terminal translocation length (bp) | Percentage of terminal translocation along left arm | Percentage of terminal translocation along right arm |
| --- | --- | --- | --- | --- | --- | --- | --- |
| AL01 | 594,546,470 | 330,424,304 | 330,424,304 | 264,122,166 | 59,507,694 | 18.01% | 22.53% |
| AL02 | 587,543,788 | 314,882,018 | 314,882,018 | 272,661,770 | 33,748,120 | 10.72% | 12.38% |
| AL03 | 587,190,583 | 267,435,302 | 267,435,302 | 319,755,281 | 47,345,005 | 17.70% | 14.81% |
| AL04 | 583,925,327 | 295,812,406 | 295,812,406 | 288,112,921 | 71,840,155 | 24.29% | 24.93% |
| AL05 | 527,343,613 | 314,532,510 | 314,532,510 | 212,811,103 | 33,460,905 | 10.64% | 15.72% |
| AL06 | 513,337,126 | 243,366,204 | 243,366,204 | 269,970,922 | 33,246,749 | 13.66% | 12.31% |
| AL07 | 453,691,697 | 282,676,230 | 282,676,230 | 171,015,467 | 63,691,606 | 22.53% | 37.24% |
| Total | 3,847,578,604 | – | 2,049,128,974 | 1,798,449,630 | 342,840,237 | 16.79% | 19.99% |
| Average | 549,654,086 | – | 292,732,711 | 256,921,376 | 48,977,177 | 18.39% | |

# Table S8. Repetitive DNA composition comparison among genomes of ALO and two Pooideae species.

## A Repetitive DNA composition of the ALO genome.

| Repeat type |  | Super family | Family | Repeat sequences (bp) | Copy number | Repeat fraction | Genome fraction |
| --- | --- | --- | --- | --- | --- | --- | --- |
| Transposable elements | | | | | | | |
| Class I (Retrotransposons) | | | | | | | |
| LTR |  | *Gypsy* | | 2,045,839,268 | 1,127,011 | 59.34% | 51.65% |
|  |  | *Copia* | | 1,035,647,971 | 575,382 | 30.04% | 26.15% |
|  |  | Unknown LTR | | 77,372,748 | 61,272 | 2.24% | 1.95% |
|  |  | Other LTR | | 213,586 | 628 | 0.01% | 0.01% |
|  | Total LTR- Retrotransposons | | | 3,159,073,573 | 1,764,293 | 91.63% | 79.76% |
| Non-LTR |  | LINE | L1 | 38,994,208 | 42,780 | 1.13% | 0.98% |
|  | Total Class I retrotransposons | | | 3,198,067,781 | 1,807,073 | 92.76% | 80.74% |
| Class II (DNA transposons)-Subclass 1 | | | | | | | |
|  |  | Tc1_Mariner | | 21,858,891 | 69,956 | 0.63% | 0.55% |
|  |  | CACTA | | 29,566,991 | 75,699 | 0.86% | 0.75% |
|  |  | Mutator | | 25,388,681 | 82,292 | 0.74% | 0.64% |
|  |  | PIF_Harbinger | | 9,194,639 | 31,402 | 0.27% | 0.23% |
|  |  | hAT | | 7,600,354 | 22,266 | 0.22% | 0.19% |
| Class II (DNA transposons)-Subclass II | | | | | | | |
|  |  | Helitron | | 43,779,456 | 117,248 | 1.27% | 1.11% |
|  | Total Class II DNA transposons | | | 137,389,012 | 398,863 | 3.99% | 3.47% |
|  | Total transposable elements | | | 3,335,456,793 | 2,205,936 | 96.75% | 84.21% |
|  | Tandem and simple sequence repeats | | | 11,144,119 | 162,888 | 0.32% | 0.28% |
|  | Other repeats | | | 100,883,895 | 369,975 | 2.93% | 2.55% |
|  | Total repetitive DNAs | | | 3,447,484,807 | 2,728,799 | 100% | 87.04% |

## B Comparison of repetitive DNA composition of ALO and two other Pooideae species genomes.

| Repeat type | Super family | Family | *Avena*  *longiglumis*  (ALO) | *Brachy-podium distachyon*  (BDI) | *Oryza*  *sativa*  (OSA) |
| --- | --- | --- | --- | --- | --- |
|  | *Gypsy* | *Retand* | 9.06% | 7.70% | 3.88% |
|  |  | *Ogre* | 1.62% | 0.00% | 1.18% |
|  |  | *Athila* | 8.10% | 0.00% | 0.45% |
|  |  | *Tekay* | 17.33% | 1.05% | 1.98% |
|  |  | *CRM* | 1.92% | 0.84% | 0.55% |
|  |  | *Reina* | 0.10% | 0.00% | 0.30% |
|  |  | *Galadriel* | 0.00% | 0.00% | 0.00% |
|  |  | Unclassified *Gypsy* | 13.51% | 3.32% | 7.41% |
|  |  | Total *Gypsy* | 51.65% | 12.91% | 15.75% |
|  | *Copia* | *SIRE* | 1.40% | 0.75% | 0.75% |
|  |  | *Angela* | 17.39% | 0.48% | 0.07% |
|  |  | *Bianca* | 0.13% | 0.39% | 0.22% |
|  |  | *Ivana* | 0.12% | 0.40% | 0.22% |
|  |  | *TAR* | 0.50% | 0.30% | 0.53% |
|  |  | *Ale* | 0.60% | 0.41% | 0.30% |
|  |  | *Tork* | 0.08% | 0.18% | 0.17% |
|  |  | *Ikeros* | 0.15% | 0.18% | 0.18% |
|  |  | *Alesia* | 0.00% | 0.07% | 0.00% |
|  |  | Unclassified *Copia* | 5.77% | 0.30% | 0.34% |
|  |  | Total *Copia* | 26.15% | 3.46% | 2.78% |
|  | *PARA-RT* | | 0.00% | 0.00% | 0.00% |
|  | *Cassandra* | | 0.00% | 0.00% | 0.00% |
|  | *Caulimoviridae* | | 0.00% | 0.06% | 0.06% |
|  | Unclassified LTR | | 1.95% | 0.68% | 0.68% |
|  | Non-*Gypsy* and non-*Copia* | | 0.01% | 0.34% | 0.34% |
| Total LTR- Retrotransposons | | | 79.76% | 17.45% | 21.07% |
| Non-LTR | *LINE* | L1 | 0.98% | 0.00% | 0.02% |
| Total Class I retrotransposons | | | 80.74% | 17.45% | 21.09% |
|  | *Tc1_Mariner* | | 0.55% | 3.96% | 7.01% |
|  | *CACTA* | | 0.75% | 2.01% | 1.66% |
|  | *Mutator* | | 0.64% | 1.55% | 3.58% |
|  | *PIF_Harbinger* | | 0.23% | 0.85% | 1.18% |
|  | *hAT* | | 0.19% | 0.38% | 1.01% |
|  | *Helitron* | | 1.11% | 9.55% | 12.03% |
| Total DNA Class II transposons | | | 3.47% | 18.3% | 26.47% |
| Total Transposable elements | | | 84.21% | 35.75% | 47.56% |
| Other repeats | | | 2.83% | 0.00% | 0.31% |
| Total Repetitive DNA | | | 87.04% | 35.75% | 47.84% |
| Genome size (Mb) | | | 3,961.00 | 271.00 | 389.00 |
| Total Class I retrotransposon length (Mb) | | | 3198.11 | 45.37 | 82.04 |
| Total Repetitive DNA length (Mb) | | | 3447.65 | 92.95 | 186.09 |
| Total non-repetitive DNA length (Mb) | | | 513.3456 | 178.05 | 202.91 |

#

# Table S9. Statistics of gene function annotation of the ALO genome.

| Databases (Abbreviation; Website) | Number | Percent (%)**^*^** |
| --- | --- | --- |
| NCBI non-redundant protein (NR; ftp://ftp.ncbi.nih.gov/blast/db) | 39,390 | 96.44% |
| NOG (Non-supervised Orthologous Groups) | 38,078 | 93.23% |
| Pfam (http://pfam.xfam.org/) | 35,573 | 87.09% |
| COG (Clusters of Orthologous Groups of proteins) | 35,460 | 86.82% |
| SwissProt (http://www.uniprot.org/uniprot/?query=*&fil=reviewed%3Ayes) | 32,099 | 78.59% |
| Gene ontology (GO; http://geneontology.org/) | 21,162 | 51.81% |
| Eukaryotic orthology groups (KOG; http://www.genome.jp/kaas-bin/kaas_main) | 20,756 | 50.82% |
| Kyoto Encyclopedia of Genes and Genomes (KEGG; http://muchong.com/html/201009/2325769.html) | 17,285 | 42.32% |
| PlantTFDB (http://planttfdb.gao-lab.org/) | 2,403 | 5.88% |
| CAZy (Carbohydrate-Active enZYmes) | 931 | 2.28% |
| Number of annotated genes* | 39,558 | 96.85% |
| Number of predicted genes | 40,845 | 100% |

* 86.82% of the annotated genes exhibited homology protein domains in COG (Clusters of Orthologous Groups of proteins) [50], and 78.59% of these genes exhibited homology protein domains in Swiss-Prot. Most of the genes were annotated with the non-redundant protein (NR) sequence database (96.44% in NCBI NR), and 93.23% of the genes were annotated in NOG (Non-supervised Orthologous Groups). 87.09% of genes were annotated with Pfam [51,52], 51.81% of the genes were classified using GO terms [53], 42.32% of the genes were annotated to known plant biological pathways based on the KEGG pathway database [54], 5.88% were annotated in PlantTFDB v.5.0 [55] and 2.28% in CAZy [56]. In addition, we predicted at least 17,712 noncoding RNAs consisting of transfer RNAs (0.0027%), microRNAs (0.0532%), and small nuclear RNAs (0.0016%).
